# Intrapartum versus postpartum insertion of intrauterine device in women delivering by cesarean section

**DOI:** 10.1186/s12884-022-04681-4

**Published:** 2022-04-28

**Authors:** Ahmed Abdel-Ghany, Eissa Khalifa, Mohamed Zeen El-Din, Emad Ibrahim, Ameer Abdallah, Mahmoud Abdel-Aziz, Mazen Abdel-Rasheed, Alaa Abdel-Azim

**Affiliations:** 1 Obstetrics and Gynecology Department, Faculty of Medicine, MINIA University, Minya, Egypt; 2 Reproductive Health Research Department, National Research Centre, 33 El-Buhouth St, Dokki, Cairo, 12622 Egypt

**Keywords:** Cesarean Section, Contraception, Failed IUD Insertion, Intrauterine Device, Uterine Perforation

## Abstract

**Background:**

The intrauterine device (IUD), being a reversible and effective contraception method, is the most widely used worldwide. This study aims to demonstrate the efficacy of IUD insertion during elective lower segment cesarean section (LSCS) versus its insertion six weeks postpartum.

**Methods:**

A cohort study was conducted on 200 women planned for elective cesarean delivery and desired IUD as a contraceptive method. They were allocated into two groups; group I, in which IUD was inserted during LSCS, and group II, in which IUD was inserted six weeks or more after LSCS. Both groups were compared regarding failed insertion, post-insertion pain, and uterine perforation. They were followed for one year for the incidence of menorrhagia, vaginal infection, IUD displacement/expulsion, missed threads, or unintended pregnancy.

**Results:**

Women in the second group showed a significantly higher incidence of failed insertion and uterine perforation than women in the first group. On the contrary, women in the first group showed a significantly higher incidence of missed threads than women in the second group. Regarding other consequences, there were no significant differences between both groups concerning menorrhagia, vaginal infection, IUD displacement/expulsion, or unintended pregnancy.

**Conclusion:**

IUD insertion during elective LSCS showed a significantly lower incidence of failed insertion and uterine perforation than its insertion six weeks postoperative.

## Background

Intrauterine devices (IUDs) are commonly used for birth control as they provide an inexpensive, long-lasting, and reversible contraception method, with a cumulative pregnancy rate of less than 1% during the first year after insertion. Besides, there are no restrictions on their use among breastfeeding and non-breastfeeding women [1]. Regarding cost–benefit analysis, IUDs can be cost-effective if given to women immediately after delivery, especially if they have no insurance [2].

Postpartum birth control has traditionally been postponed until 6 weeks after delivery. Therefore, women have been instructed to avoid intercourse during these 6 weeks. However, some women have sexual activity early within this period, especially those who deliver by a cesarean section rather than vaginal delivery. Consequently, this six-week delay in starting a contraception method carries a high risk of unintended immediate postpartum pregnancy [3]. It also must be taken into consideration that ovulation starts early during the fourth week after delivery in non-breastfeeding women, which further increases the risk of unintentional very early postpartum pregnancy [4].

Immediate post placental IUD insertion is defined as IUD insertion within 10 min following delivery. IUD insertion within this period has the advantages of less discomfort as well as increased motivation for contraception [5]. Several studies have investigated the immediate post placental IUD insertion regarding safety, effectiveness, and expulsion rates. Generally, immediate post placental IUD insertion during cesarean delivery has an expulsion rate of less than 14% [6].

In this study, we aimed to compare IUD insertion during lower segment cesarean section (LSCS) versus planned interval IUD placement 6 or more weeks post-cesarean delivery.

## Methods

After having the approval of the Medical Research Ethics committee of Minia University, we conducted this cohort study at the Maternity Hospital of Minia University starting from November 2017 till October 2019. All participants gave their consents after being clearly informed about the study's objective and design, giving them the option of leaving the study at any time.

The study population included 200 pregnant women who were referred from the antenatal clinic to undergo elective cesarean delivery and who desired to use the copper IUD for postpartum contraception. These 200 women were allocated into two groups; group I, in which women chose to insert the IUD during their LSCS, and group II, in which women chose to insert the IUD during their postnatal follow-up visit (six weeks after LSCS).

We included pregnant women with previous cesarean deliveries who did not experience vaginal delivery before. The gestational age was between 38–40 weeks at the time of enrollment, and they had normal ultrasound findings regarding gestational age, uterine cavity, and placental site (should be away from the scar). Women were excluded if they had distorted uterine cavity or any uterine anomalies, uterine myoma, suspected cervical or uterine neoplasia, known allergy to copper, history of ruptured membrane for more than 12 h, history of ectopic pregnancy, history of repeated pelvic infections, or history of menorrhagia, coagulopathy, or severe dysmenorrhea.

In group I, the copper IUD was inserted during cesarean section just after delivery of the baby, placenta, and membranes. The IUD included in its plastic sleeve was placed at the fundus of the uterine cavity, and its threads within the plastic sleeve were negotiated through the cervix after its dilatation by the tip of a finger. After the uterine and abdominal walls were closed, the plastic sleeve was gently removed through the vagina, and the threads were shortened 2 cm below the external cervical os level. On the other hand, women of the second group underwent routine IUD insertion during their postnatal follow-up visit (six weeks after LSCS).

After IUD insertion, all women underwent routine postnatal follow-up for one year, including medical history, vaginal examination, and transvaginal ultrasound. IUD was considered displaced if the distance between the fundal endometrial surface and the IUD was more than 3 mm after uterine involution, while expulsion was considered if the IUD passed either partially or wholly through the external cervical os.

Both groups were compared regarding immediate post insertion sequelae, including failed IUD insertion, post-insertion pain, and uterine perforation. In addition, all women were followed up for possible remote IUD complications, such as menorrhagia, vaginal infection, IUD displacement/expulsion, missed threads, and unintended pregnancy.

### Sample size calculation

According to a previous study, the proportion in group one (the treatment group) is assumed to be 64% under the null hypothesis and 83% under the alternative hypothesis [7]. The proportion in group two (the control group) is 64%. Therefore, the sample size of 83 in each group achieve 80% power with a significance level of 0.05 to detect a difference between the groups. We increased the sample size by 20% to be 100 in each group for dropout.

### Statistical analysis

Data were coded and analyzed using the statistical package for social science; (SPSS Inc., Chicago, IL, USA, v.25 for Microsoft Windows). Statistical analysis was done to obtain the mean ± standard deviation (SD), median, and range for numerical variables, and frequencies and percentages for categorical variables. A Student t-test and Mann–Whitney test were used for numerical data, while a Chi-square test was used for categorical data. A *p*-value < 0.05 was considered statistically significant.

## Results

**Fig. 1:**
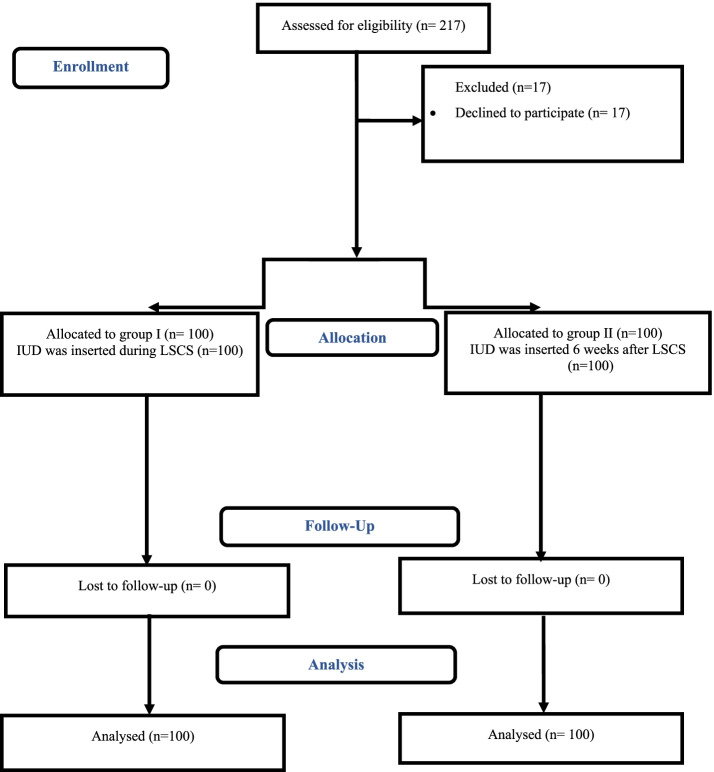
Flowchart of participants in the study

This study included 200 women seeking IUD as a contraception method after elective cesarean delivery and fulfilling the inclusion criteria as shown in Fig. 1. The demographic and obstetric data of women in both groups were demonstrated in Table 1. There were no significant differences between women in both groups regarding age, body mass index (BMI), parity, number of previous abortions, years after the last delivery, and gestational age (at the time of current delivery).

**Table 1:** Demographic data of both groups

	**Group I**	**Group II**	***P*-value**
	**(*n* = 100)**	**(*n* = 100)**	
**Age (year)**	31.66 ± 3.50	32.06 ± 3.26	0.404 (NS)
**BMI** (kg/m^2^)	28.63 ± 2.34	29.16 ± 3.31	0.199 (NS)
**Parity**	2 (1–3)	2 (1–3)	0.289 (NS)
**Previous abortions**	1 (0–2)	1 (0–3)	0.609 (NS)
**Time since last delivery (years)**	2 (1–3)	2 (1–4)	0.371 (NS)
**GA at current delivery (weeks)**	39.15 ± 0.74	39.19 ± 0.69	0.694 (NS)

*BMI* Body mass index, *GA* Gestational age, *NS* Not significant (*p* > 0.05)

Table 2 demonstrates the consequences that occurred following IUDs insertion in women of both groups. Women in group II showed a significantly higher incidence of failed IUD insertion and uterine perforation than women in the first group (failed IUD insertion accounts for 6% vs. 0%, *P* = 0.013, while uterine perforation accounts for 5% vs. 0%, *P* = 0.024). On the other hand, the incidence of missed threads was significantly higher in women of the first group (13%) than women in the second group (4%) by the end of the follow-up (*P* = 0.04). Regarding other consequences assessed during the follow-up period (one year), there was no significant difference between both groups concerning menorrhagia, vaginal infection, IUD displacement, and IUD expulsion. Both groups had no single case of unintended pregnancy during the first year following IUD insertion.

**Table 2:** Consequences of IUD Insertion in both groups

	**Group I**	**Group II**	***P*-value**
	**(*n* = 100)**	**(*n* = 100)**	
**Failed insertion**	0 (0%)	6 (6%)	0.013*
**Post insertion pain**	11 (11%)	15 (15%)	0.4 (NS)
**Uterine perforation**	0 (0%)	5 (5%)	0.024*
**Menorrhagia**	17 (17%)	24 (24%)	0.22 (NS)
**IUD Displacement**	10 (10%)	5 (5%)	0.179 (NS)
**IUD Expulsion**	5 (5%)	2 (2%)	0.248 (NS)
**Missed threads**	13 (13%)	4 (4%)	0.04*
**Vaginal Infection**	0 (0%)	1 (1%)	0.316 (NS)
**Unintended Pregnancy**	0 (0%)	0 (0%)	NA

*IUD* Intrauterine device; *: significant (*p* ≤ 0.05); NS: not significant (*p* > 0.05)

## Discussion

The copper IUD has been considered as an appropriate contraception method for almost all women. This study aimed to compare copper IUD insertion immediately after placental expulsion during LSCS versus interval IUD insertion regarding efficacy, safety, convenience, and complications.

Mohamed et al. (2003) investigated the factors that affected postpartum IUDs insertion among the population of Assiut governorate, Egypt. They concluded that IUD acceptance as a contraceptive method was lower than predicted, and actual insertion was much worse. For those women, the only time they could get details about birth control methods was when they approached medical providers during delivery. As a result, it was recommended that family planning programs should be combined with maternity and childbirth services. This would provide better encouragement for the use of contraception devices in women who would not search them out on their own [8].

We found no significant difference between immediate IUD insertion following cesarean delivery and interval IUD insertion regarding failure rate or occurrence of unintended pregnancy. These results are in agreement with the World Health Organization, which has classified the copper T380A IUD as category 1 medical eligibility for contraception when used early in the immediate postpartum period. It has been demonstrated that immediate postpartum IUD insertion is a secure alternative for delayed postpartum insertion [9].

Furthermore, we found no significant difference between both study groups regarding IUD displacement and expulsion rate. The feasibility of immediate postpartum IUD insertion is supported by its popularity in diverse countries such as Mexico, China, and Egypt. This technique has many advantages, including increased incentive, confidence that the woman is not pregnant, and comfort. Early follow-up is essential for detecting spontaneous IUD expulsion. Levi et al. (2012) investigated the expulsion rate of copper T380A IUDs inserted during cesarean delivery. They assessed the feasibility of enrolling and following women who chose to insert an IUD immediately after cesarean delivery. They used a cohort study to gather data regarding IUD expulsion rates and observed that women accepted imminent post-placental IUD insertion at the time of cesarean delivery as they thought it was secure and satisfactory [10].

On the contrary, Eroglu et al. (2006) assessed the effectiveness and risks of immediate post-placental, early postpartum, and interval IUD insertions. They demonstrated that immediate post-placental and early postpartum insertion groups showed more complications as well as higher expulsion rates than the interval IUD insertion group [11]. Similarly, a meta-analysis in 2015, including only 4 studies, revealed that the IUD expulsion rate within six months was more likely for the immediate post-placental IUD group, but the confidence interval was wide (OR 4.89, 95% CI 1.47—16.32) [12].

In 2009, Kapp and Curtis also reported that the expulsion rate of IUD inserted immediately postpartum after vaginal delivery is higher than that for interval insertion [13]. However, Kapp and Sonalkar updated this prior systematic review about IUD insertion safety and expulsion rates during cesarean delivery [14]. A broader study, including multiple randomized controlled trials of IUD insertion during cesarean delivery, is required to further assessment of the expulsion rates and the continuation rates associated with IUDs placed at the time of cesarean delivery.

There is a disparity in IUD expulsion rates between vaginal and cesarean deliveries, which may be explained by the fact that the cervix is usually not fully dilated during cesarean delivery, making IUD expulsion into the cervical canal more difficult. Furthermore, since the entire uterus can be easily seen, palpated, and examined during cesarean delivery, it is theoretically easier to achieve sufficient fundal positioning of the IUD after expulsion of the placenta.

The most common side effects of the copper IUD are menorrhagia and dysmenorrhea. Hubacher et al. (2009) reported that 53% of women complained of these symptoms. However, none of them said they were unhappy with the IUD in general, and none of them decided to remove it [15]. In our study, there was no significant difference between both groups concerning post insertion pain or bleeding,

Bhutta et al. (2011) examined the safety of Multiload Cu 375 IUD insertion during the cesarean section after placental expulsion regarding infection, unintended pregnancy rate, and uterine perforation. They also compared women who had IUD insertion during cesarean section with women who had IUD interval insertion regarding pain, amount of bleeding, and expulsion rate. They concluded that women who would deliver by a cesarean section and who are willing and prepared to use the IUD for contraception should be given the option of IUD insertion at the same time of their cesarean delivery [16]. These results were concomitant with our study.

In the same context, Çelen et al. (2011) investigated the effectiveness and safety of inserting TCu 380A IUD just after placental expulsion during the cesarean section. The primary outcome indicators were the 12-month cumulative rates of unintended conception, IUD expulsion, and medically related IUD complications. They revealed that placement of IUD during a cesarean section just after placental expulsion offers sufficient pregnancy protection without increased risk of infection [5], which agrees with our results. Our study demonstrated that there was no significant difference between women with immediate IUD insertion during cesarean delivery (group 1) and women with interval IUD insertion (group 2) regarding infection rate, which was 0% in group 1 versus 1% in group 2.

However, Çelen et al. (2011) observed that one out of every four women discontinued IUD use either due to expulsion or any other medical reason [5]. This contradicts some other studies which claimed improved tolerance of immediate IUD insertion after cesarean section. In certain circumstances, IUD insertion during cesarean deliveries immediately after expulsion of the placenta can still be an option. However, considering the high incidence of IUD expulsion, regular assessment of the proper positioning of the IUD during the first year, as well as periodic follow-up visits in subsequent years, are highly recommended [5].

The main limitation of the current study is that the follow-up period of the participant women was short (one year only) and was not at a certain scheduled time frame. In addition, during follow-up, women who experienced IUD expulsion or even displacement preferred to shift to another contraception method that affected unintended pregnancy data. Furthermore, IUD post-insertion pain in the first group possibly had been masked by the postoperative analgesics. Interestingly, the incidence of missed threads was significantly higher in women of the first group than women in the second group by the end of the follow-up. This could be attributed to leaving the threads longer upon insertion and shortening the threads at a follow-up screening visit.

## Conclusion

There is worldwide debate regarding the value of IUD insertion during elective lower segment cesarean section (LSCS), especially in women with one or more previous cesarean sections. We concluded that there is no significant difference between IUD insertion during elective LSCS and its insertion six weeks later regarding the consequences of both methods, such as menorrhagia, vaginal infection, IUD displacement/expulsion, or unintended pregnancy. On the other hand, IUD insertion during elective LSCS showed a significantly lower incidence of failed insertion and uterine perforation than the interval insertion.

## Data Availability

The datasets used and/or analyzed during the current study are available from the corresponding author on reasonable request.
